# *Bacillus velezensis* QA2 Potentially Induced Salt Stress Tolerance and Enhanced Phosphate Uptake in Quinoa Plants

**DOI:** 10.3390/microorganisms10091836

**Published:** 2022-09-14

**Authors:** Ismail Mahdi, Abdelmounaaim Allaoui, Nidal Fahsi, Latefa Biskri

**Affiliations:** 1 Microbiology Laboratory, Mohammed VI Polytechnic University, Lot 660, Hay Moulay Rachid, Ben Guerir 43150, Morocco; 2 African Genome Center (AGC), Mohammed VI Polytechnic University, Lot 660, Hay Moulay Rachid, Ben Guerir 43150, Morocco

**Keywords:** *Chenopodium quinoa*, phosphate solubilizing bacteria, plant growth promotion, biocontrol, *Bacillus velezensis*, salt stress

## Abstract

Plant Growth-Promoting Rhizobacteria (PGPR) have attracted much attention in agriculture biotechnology as biological inputs to sustain crop production. The present study describes a halotolerant phosphate solubilizing bacterium associated with quinoa plant roots. Based on a metabolic screening, one bacterial isolate, named QA2, was selected and screened for PGPR traits. This isolate solubilized both inorganic phosphate and zinc, produced indole-3-acetic acid, ammonia, hydrogen cyanide, cellulase, and (to be deleted) protease, and induced biofilm formation. We demonstrated that QA2 exhibited both antimicrobial and ion metabolism activities and tolerated high salt concentration at up to 11% NaCl. Genotyping analyses, using *16S rRNA* and chaperonin *cpn60* genes, revealed that QA2 belongs to the species of *Bacillus velezensis*. Using the quinoa model cultivated under a saline condition, we demonstrated that QA2 promoted plant growth and mitigated the saline irrigation effects. Analysis of harvested plants revealed that QA2 induced a significant increase of both leaf chlorophyll index by 120.86% (*p* < 0.05) and P uptake by 41.17% (*p* < 0.05), while the content of Na^+^ was drastically decreased. Lastly, a bibliometric data analysis highlighted the panoramic view of studies carried out so far on *B. velezensis* strains. Our investigation presents a holistic view of the potential application of *B. velezensis* as a biological inoculant to promote plant growth, control pathogen attacks, and mitigate the salinity effect of quinoa plants. Further investigations are still needed to demonstrate these effects in field conditions.

## 1. Introduction

The development of sustainable and efficient agriculture needs to find new solutions to enhance the salt tolerance characteristics of crops. Plant Growth Promoting Bacteria (PGPR) represent a promising solution to strengthen agricultural productivity. They are expected to provide new solutions to improve the salt tolerance characteristics of plants. Indeed, they improve the availability of phosphorous and nutrients to crops, improve soil structure, and promote healthy and fertile soil [1,2,3]. Several strategies have been established to reduce the damaging effects of salt stress on plants both by genetic engineering and using PGPR [4]. Microorganisms colonizing the rhizosphere and inhabiting the plant tissues stimulate plant growth via several indirect and direct mechanisms [5]. Other studies tried to identify appropriate biofertilizers such as *Bacillus*-based biofertilizers that display high resilience to diverse environmental stresses because of their ability to form spores. *Bacillus* strains are more suitable as biofertilizers due to their production of metabolites that confer more qualities than the non-spore-forming *Pseudomonas* strains [6]. Some examples of *Bacillus*-based biofertilizers include Alinit, Kodiak (*Bacillus subtilis* GB03), Quantum-400 (*B. subtilis* GB03), Rhizovital (*B. amyloliquefaciens* FZB42), and YIB (*Bacillus* spp.) [7,8,9]. However, none of these commercialized biofertilizers confer simultaneous efficient use of P, tolerance to salt, and drought stress of crops. Thus, there is an urgent need to develop new biofertilizers to help increase crop resilience to multiple biotic and abiotic stresses.

Salinity is aggravated by the exploitation of agricultural lands in urban areas, increasing the competition between crops and energy plant species. Indeed, salt stress is a major encountered issue that affects crop production. Saline and dry soils are poorly accessible for agricultural cultivation. Nearly 10% of the land surface and 50% of total irrigated land in the world are affected by salinity, causing annual losses in agricultural production that are more than 12 billion US$ [10,11]. Salinity negatively influences the physicochemical properties of soils and suppresses the growth of both plants and soil microbes.

Several approaches have been developed to reduce the toxic effects caused by high salinity on plants. These include the use of conventional breeding, halophyte varieties, and transgenic editing. However, these strategies are time-consuming, unsustainable, and labor-intensive [12]. In this context, many investigations reported that in addition to the inherent mechanisms of plants to cope with salt stress, plant-associated microorganisms can be harnessed to promote plant performance and yield under such conditions. Thus, the bioremediation of saline soils requires the use of plants and microbes that can tolerate salinity. There is a requirement to increase global food production by about 70% to meet the 2050 challenge of feeding 9.3 billion people [13]. In fact, salt tolerant PGPB mediates several mechanisms within plants facing salinity. These include exopolysaccharides production, osmoprotectants synthesis, antioxidant enzyme production, phytohormone production, and 1-aminocyclopropane-1-carboxylic acid (ACC) deaminase activity [12,14]. This enhances nutrient assimilation, promotes homeostasis, and increases antioxidant response during salty conditions.

Only a few studies have addressed halotolerant PSB associated with the pseudo-cereal quinoa plants. Quinoa natively grows in the Andean region, at >4000 m above-sea-level and is considered the traditional crop in South America. In the recent past, quinoa has gotten increasing attention globally, due to its exceptional nutritional properties. The seeds are gluten-free and rich in minerals, proteins, and vitamins [15,16]. Quinoa is a facultative halophyte, able to cope with high levels of salinity and drought stress. Some varieties even can be irrigated by sea water [17].

Here, we isolated a new halotolerant PSB strain (QA2) from quinoa fields in Morocco. We demonstrated that the resulting isolate QA2 belongs to the species of *Bacillus velezensis* and enhances plant salt tolerance and phosphorus use efficiency in vivo.

## 2. Materials and Methods

### 2.1. Soil Sampling

A total of 12 rhizosphere soil samples of 3-month-old quinoa plants were collected from quinoa fields of the experimental farm (32.219731E, −7.892268N) of Mohammed VI Polytechnic University—Ben Guerir, Morocco. The rhizosphere soils were sampled in June, the peak growing season for quinoa. The soil samples were collected within 20–30 cm of quinoa plants. Samples including roots and soil aggregates adhering to the roots weighing approximately 50 g were taken from each site, placed individually in sterile plastic bags, and immediately stored at 4 °C in a cooler and transported to the laboratory. All samples were stored at 4 °C until use [18].

### 2.2. Isolation, Screening, and Purification of Phosphate Solubilizing Rhizobacteria on Plates

Bacteria were isolated from collected samples by uprooting the remaining parts of the quinoa plants, and the soils attached to the roots were separated by a scalpel [19]. One g of soil from each sample was aseptically introduced into a tube containing 9 mL of sterile distilled water and then vigorously shaken for 30 min. Afterward, a series of decimal dilutions (10^−1^ to 10^−6^) of the suspensions were carried out in Eppendorf tubes using sterilized distilled water. To isolate potential PSB following the pour plate technique, 100 μL of each dilution were plated on TSA (Trypticase Soy Agar) (VWR, Casablanca, Morocco) nutrient agar following the standard spread plate technique. A total of 79 bacterial isolates exhibiting distinguishable morphological aspects were spot-inoculated on NBRIP (National Botanical Research Institute’s phosphate) agar medium plates consisting of dextrose 10 g; Tricalcium Phosphate (TCP) as Ca_3_(PO_4_)_2_, KT) 5 g (purum p.a., ≥90% (Sigma-Aldrich, Overijse, Belgium); ammonium sulfate (NH_4_)_2_SO_4_) 0.5 g/L; potassium chloride (KCl) 0.2 g/L; sodium chloride (NaCl) 0.2 g/L; magnesium sulfate (MgSO_4_) 0.1 g/L; ferrous sulfate (FeSO_4_) trace; manganese sulfate (MnSO_4_) trace; and agar 15 g (VWR, Casablance, Morocco); the pH was adjusted to 6.75 ± 0.25 before autoclaving [20]. Plates were incubated at 30 °C and observed daily for 7 days for the formation of transparent halo zones around the colonies. The halo around the bacterial colony indicated P-solubilizing capacity. Colonies showing discrete hallo zones were purified on the same medium and stored at −80 °C in cryotubes in 10% Dimethyl sulfoxide (DMSO) until use.

### 2.3. Screening for Salt Tolerance

To assess the salinity tolerance of the isolated PSB strains, bacteria were streaked on TSA plates supplemented with various NaCl concentrations (0–14%) (*w*/*v*) and incubated for 48 h at 30 °C. To estimate the maximal growth and minimal inhibitory salt concentrations (MIC), selected bacteria were further tested in liquid TSB (Tryptone Soy Broth) (BIOKAR Diagnostics, Allonne, France) using 48-well microtiter microplates. Briefly, 2 μL of the bacterial cultures (OD_600nm_ = 0.8) were inoculated in 500 μL TSB with increasing NaCl concentrations (0, 2, 4, 6, 8, 10, 12, 14, 16, 18%) (*w*/*v*) and incubated at 30 °C under shaking at 150 rpm. Growth patterns were evaluated using the VICTOR Nivo^TM^ Multimode Plate Reader (HTDS, Casablanca, Morocco) at 600 nm following 48 h of incubation [21,22]. Based on this assay, one isolate named QA2 was selected for downstream characterization. Our published strain QF11 (*Enterobacter asburiae*) was used as a reference PGPR as we and others have shown that it is a potent bioinoculant and biocontrol agent of various crops [23,24,25,26].

The QA2 isolate was also examined for its ability to tolerate heat stress. In brief, we streaked the bacterium on TSA plates and incubated them at different temperatures ranging from 30 to 60 °C. Post 24 h of incubation, the thermotolerance of the bacterial isolate was determined by observing its growth [27].

### 2.4. Quantitative Assay of P Solubilization in Liquid Media

The selection of P-solubilizing bacteria based on plates is a relative efficiency test and is not an indicative method of identifying whether a strain is a P-solubilizer [28]. Thus, P solubilization activity was next quantified using insoluble TCP as a sole source of phosphate in the NBRIP broth medium [29]. The isolate was grown overnight in TSB broth and the optical density (OD_600nm_) was adjusted to 0.8. Bacterial suspensions (100 μL) were inoculated in 250 mL vials containing 50 mL of NBRIP broth. After 5 days of incubation at 30 °C under shaking at 150 rpm, the cultures were centrifuged at 12,000 rpm for 10 min and the supernatants were collected and filtered through 0.22 μm sterile syringe filters to remove insoluble materials [30]. The cell-free supernatants were diluted (1/50) and used to calorimetrically measure the soluble P content at the agricultural innovation and technology transfer center (AITTC–UM6P) using a Continuous Flow Analyzer (SKALAR SAN^++^ SYSTEM, Casablanca, Morocco). Uninoculated flasks containing an un-inoculated NBRIP medium served as a control. The experiment was performed in triplicate and the results are reported as mean ± standard deviation for each sample. The final pH of the supernatants was also measured.

### 2.5. DNA Amplification and Phylogenetic Identification of Selected Rhizobacterium

The taxonomic identification of selected bacterium was carried out using the 16S rRNA and the chaperonin-60 (cpn60) gene DNA sequencing. Primers pA (5′–AGAGTTTGATCCTGGCTCAG–3′) and 926R_Quince (5′–CCGYCAATTYMTTTRAGTTT–3′) were used to amplify the *16S rRNA* gene, while degenerated primers H279 (5′–GAIIIIGCIGGIGAYGGIACIACIAC–3′) and H280 (5′–YKIYKITCICCRAAICCIGGIGCYTT–3′), with modified 5′-ends, were used for *cpn60* gene amplification [31]. All PCR amplifications were carried out in 50 μL reactions containing: 23 μL water, 25 μL MyTaq Mix, 1 μL of the forward and reverse primers (20 µM final concentration), and 1 μL of fresh overnight bacterial culture as a DNA matrix. The PCR profile included an initial heat step of 94 °C for 5 min, followed by 35 cycles of denaturation at 94 °C for 30 s, annealing at 54 °C for 30 s, and extension step at 72 °C for 1 min, and final extension at 72 °C during 10 min. Used primers for *16S rRNA* gene-targeted variable regions V1–V5. The amplified DNA PCR products (~900 bp for *16S rRNA* sequences, and ~600 bp *cpn60* amplicons) were commercially sequenced. Generated DNA sequences were analyzed using UGENE (Unipro UGENE: a unified bioinformatics toolkit Konstantin Okonechnikov, Olga Golosova, Mikhail Fursov, the UGENE team. Bioinformatics 2012 28: 1166-1167.doi: 10.1093/bioinformatics/bts091) and SILVA (https://www.arb-silva.de (accessed on 20 November 2020) program (for 16S rRNA sequences) and alignment search using BLAST stool of at NCBI (for 16S rRNA and cpn60 sequences) databases [32]. The *16S rRNA* gene sequence was deposited in the NCBI GenBank and the sequence accession number was provided. The phylogenetic tree was built using the neighbor-joining method in SILVA, downloaded, and annotated in Interactive Tree Of Life (iTOL) [33].

### 2.6. In Vitro Evaluation of PGP Traits

#### 2.6.1. Indole-3-Acetic Acid (IAA) Production Assay

Quantitative estimation of IAA produced by bacterial isolate was estimated in a TSB medium supplemented with L-tryptophan as a precursor of IAA [34]. Bacteria were cultured in 50 mL of TSB broth supplemented with 0.1% L-tryptophan and incubated at 28 ± 2 °C in a shaking incubator at 150 rpm for 7 days [35]. The bacterial cultures were subsequently centrifuged at 12,000 rpm for 10 min at 4 °C and the supernatants were filtered through 0.22 μm filters. Two mL of *Van Urk Salkowski* reagent consisting of 1 mL of 0.5 M FeCl_3_ and 50 mL of 35% *v*/*v* HClO_4_ (Merck, Darmstadt, Germany) were mixed with 1 mL of each filtrate. After incubation in the dark for 30 min at room temperature, the development of a pinkish color indicated the production of IAA. Absorbance was then measured at 535 nm using a spectrophotometer (UV/VIS Scanning Spectrophotometer, Casablanca, Morocco) [35]. The amounts of IAA produced were determined according to a standard curve of pure IAA (Sigma Aldrich, Overijse, Belgium) for concentrations in the 0–100 µg/mL range.

#### 2.6.2. Biofilm Formation Assay

The biofilm formation was assessed according to the quantitative colorimetric assay [36]. Fresh overnight bacterial culture was diluted to 1/100 in TSB medium (BIOKAR Diagnostics, France) and 200 μL of the bacterial suspension (OD_600nm_ = 0.8) were inoculated in triplicate into a 48-well microplate. Uninoculated TSB was used as a negative control. The microplate was incubated at 30 °C for 24 h. Next, the bacterial culture was centrifuged, the supernatant was discarded, and the bacterial pellet was washed three times with 200 μL of the buffer solution (PBS) to remove unattached planktonic bacteria. Biofilm formation was monitored by adding 1% crystal violet (Merck, Darmstadt, Germany) solution for 15 min at room temperature. The excess dye was removed by washing three times with distilled water. The bacterial biofilm was then solubilized using 200 μL of 95% ethanol and the OD_600nm_ was measured using the VICTOR Nivo^TM^ Multimode Plate Reader (PerkinElmer, Casablanca, Morocco). The absorbance values were considered as an index of bacteria forming biofilms. Uninoculated medium and *Enterobacter asburiae* QF11 served as negative and positive controls, respectively.

#### 2.6.3. Qualitative Siderophores Production Assay

Qualitative assessment of the siderophore-producing capacity of the selected PSB was performed using the universal Chrome Azurol S (CAS) agar plate assay according to the method of [37]. Four solutions were used: Fe-CAS indicator (solution1), buffer (solution 2), mixing solution (solution 3), and casamino acid (solution 4) (Fisher Scientific, Illkirch-Graffenstaden, France) [38]. To prepare the Fe-CAS indicator solution, 0.06 g CAS was dissolved in 50 mL distilled water and mixed with 10 mL iron solution (1 mM FeCl_3_-6H_2_O, 10 mM HCl) (VWR Chemicals, Casablanca, Morocco). Under the stirring condition, this solution was slowly added to 0.073 g HDTMA (hexadecyltrimethylammonium bromide) dissolved in 40 mL distilled water. The obtained dark blue liquid was autoclaved and cooled. To prepare buffer solution, 32.24 g PIPES (Piperazine-N, N’-bis (2-éthanesulfonique) (VWR Chemicals, Radnor, PA, USA) was dissolved in 7.5% (*v*/*v*) of mixing salt (solution3) containing (in 100 mL distilled water) 3 g KH_2_PO_4_, 5 g NaCl, and 10 g NH_4_Cl. The pH of the PIPES was raised to 6.8 using a KOH solution. Buffer solution (2) was autoclaved after adding 15 g of agar and then cooled to 50 °C. To prepare the casamino acid solution, 3 g of casamino acid was dissolved in 27 mL of distilled water and sterilized using 0.22 µm filters. The four solutions were vigorously mixed and poured into Petri dishes. After solidifying, Chrome Azurol S agar plates were spot inoculated with each isolate, and the plates were then incubated for 7 days at 30 °C. Bacteria-producing siderophore form orange halo around colonies due to iron chelation. Results were visually analyzed for halo formation against the blue medium. The experiment was performed in triplicate.

#### 2.6.4. Ammonia Production Assay

The ammonia production activity of bacteria was performed as previously described [39]. A bacterial suspension (100 μL (OD_600nm_ = 0.8)) was inoculated into tubes containing 10 mL of sterile peptone water (peptone 10 g; sodium chloride 5 g and distilled water 1 L and pH was adjusted to 6.75 ± 0.25 and incubated at 30 °C with shaking at 150 rpm for 96 h. Uninoculated medium and QF11 isolate served as negative control (C−) and positive controls, respectively. Afterward, aliquots (1 mL) were taken and centrifuged at 10,000 rpm for 10 min. Then, 0.5 mL of Nessler’s reagent (VWR Chemicals, Rosny-sous-Bois cedex, France) was added to each aliquot. The ammonia production was considered positive following the development of a brownish coloration and absorbance was measured at 450 nm using a spectrophotometer. Ammonia produced was estimated using a standard curve of ammonium sulfate for concentrations in the 0–0.3 µmol/mL range [40].

#### 2.6.5. Zinc Solubilization Assay

The bacterial ability to solubilize Zinc was evaluated using Tris-mineral agar medium containing 1 L of distilled water: D-glucose 10.0 g, (NH_4_)_2_SO_4_ 1.0 g, KCl 0.2 g, K_2_HPO_4_ 0.1 g, MgSO_4_ 0.2 g (Scharlau, Casablanca, Morocco), pH = 6.75 ± 0.25. The media were amended with three different sources of insoluble zinc compounds (Sigma-Aldrich) mainly zinc oxide (ZnO) (15.23 mM), zinc phosphate (Zn_3_(PO_4_)_2_) (5.0 mM), and zinc carbonate (ZnCO_3_) (5.2 mM) at a 0.1% Zn final concentration [41]. The selected bacterium was spotted on each Tris-mineral medium and plates were incubated at 30 °C for 10 days. Zinc solubilizers showed a clear halo zone around the colony. The experiment was performed in triplicate.

#### 2.6.6. HCN Production Assay

Bacteria were tested for hydrogen cyanide (HCN) production by adapting the method of Lorck (1948) [42]. Briefly, the TSA medium was amended with 0.44% glycine (VWR Chemicals, China) and 100 μL (OD_600nm_ = 0.6) of the bacterial culture was flooded on poured agar plates with a sterile swab. A Whatman filter paper was soaked in the picric acid (0.5%) in 2% sodium carbonate for 1 min and placed below the plates’ lids. The plates were closed with parafilm and incubated at 30 °C for 96 h. The color shift of Whatman paper from yellow, conferred by sodium picrate solution, to orange or brown indicates a positive result for HCN production. The experiment was performed in triplicate.

#### 2.6.7. Extracellular Enzymes Production Assay

Bacteria were analyzed to produce proteases and cellulase. The proteolytic activity was qualitatively assessed according to the previously reported method using a medium having the following composition (in 1 L of distilled water): 5 g pancreatic casein (Merck, Darmstadt, Germany); 2.5 g yeast extract; 1 g glucose, and 15 g agar. The pH of the medium was adjusted to 6.75 ± 0.25 before autoclaving. After cooling, the medium was amended with 100 mL of a 10% sterilized skim milk solution and seeded by the spot inoculation method. Halos of paracasein precipitation around colonies appearing over the next 48 h were considered evidence of positive proteolytic activity [43].

As for the cellulase activity, bacteria were incubated on a mineral salt agar plate containing 0.4% (NH_4_)_2_SO_4_, 0.6% NaCl, 0.1% K_2_HPO_4_, 0.01% MgSO_4_, 0.01% CaCl_2_ with 0.5% carboxymethyl cellulose (CMC) (Merck, Germany), and 2% agar. Once the incubation was achieved, a solution of 1% Congo red (Labo Chemie, Mumbai, India) was added to the surface of grown cultures for 20 min. Next, the surfaces of the plates were flooded with a solution of 1 M NaCl and left to stand for 30 min. The appearance of a clear halo around the colonies indicates the degradation of the CMC and reflects bacterial extracellular cellulase production [44]. The experiments were carried out in triplicate and the diameters of the halos were measured in centimeters.

#### 2.6.8. Antibacterial Activity Assay

Antibacterial activity was performed using the well-diffusion method previously described [45]. Six clinical isolates pathogenic strains, kindly provided from the microbiology laboratory of the faculty of medicine (FMPM)—Marrakech, Morocco), were used in this assay namely the bacterium *Staphylococcus aureus*, *Salmonella typhi*, *Enterococcus faecalis*, and *Escherichia coli* and the fungus *Candida albicans*. The surface of the agar plate (Mueller–Hinton agar for bacteria and potato dextrose agar (PDA) for the fungus) was inoculated by spreading 100 μL from overnight cultures of each pathogenic strain (OD_600nm_ = 0.6 for bacteria, OD_530nm_ = 0.3 for *C. albicans*) [46]. Next, filter papers discs 6 mm in diameter, previously soaked in 30 μL of the filtered bacterial cell-free supernatant, were deposited on the plate surfaces and incubated for 24 h at 37 °C in the case of bacteria, and for 48 h at 25 °C for *C. albicans* [47]. The appearance of an inhibition growth zone was taken as evidence of antimicrobial activity. The experiments were carried out in triplicate.

#### 2.6.9. Antifungal Activity Assay

Bacterial isolate was tested in an in vitro preliminary assay to assess its potential antagonistic effect against *Fusarium oxysporum f.sp. albedinis* growth, kindly provided from ESAFE school (Mohammed VI Polytechnic University, Ben Guerir, Morocco). A loop of bacterial cells from fresh overnight culture was streaked on PDA plates (Biokar Diagnostics, Allonne, France). Then, a fungal spot was placed at the same distance from the bacterial line. Plate-containing fungus only was used as a control. Plates were sealed with parafilm, and incubated at room temperature for 4 days, and the growth of the fungus was daily observed. *Escherichia coli* (*E. coli*) DH5α was used as a control species because we have its complete genome annotation, and it is usually used as a bacterial strain control.

#### 2.6.10. Bacterial Antibiotic Resistance Pattern

Briefly, freshly prepared TSA plates were amended, after cooling to less than 50 °C, with prepared concentrations of six frequently used antibiotics namely Ampicillin (100 µg/mL), Kanamycin (50 µg/mL), Tetracycline (10 µg/mL), Streptomycin (100 µg/mL), Chloramphenicol (20 µg/mL), and Spectinomycin (60 µg/mL) (Merck, Darmstadt, Germany). Plates were streaked with overnight grown bacterial cultures. The resistance profiles were determined by checking bacterial growth after 24 h of incubation at 30 °C.

### 2.7. In-Vivo Inoculation Experiments

#### 2.7.1. Seed Germination Assay

Quinoa seeds were firstly sorted and selected to discard those with abnormal or damageable aspects. Next, all seeds were surface sterilized with 70% ethanol for 1 min, shaken in 2% sodium hypochlorite solution for 1 min, and washed five times in sterilized distilled water for 15 min followed by air-drying under a laminar flow hood. As a bioinoculant, an OD_600nm_ of 0.8 of the strain was used. The cell pellet of bacterial suspension was obtained from a fresh overnight culture by 5 min centrifugation at 10,000 rpm. The bacterial pellet was resuspended in 10 mL of distilled sterile water and vortexed for 10 s before being used for seed treatment. The bacterial suspension was applied as seed drenches with a ratio of 10 mL per 90 seeds for 1 h [48]. Afterward, seeds were air-dried and placed on sterilized Petri dishes containing 0.7% agar [49]. A total of 30 seeds were placed on each plate. Triplicates of the treatment were maintained, and seeds treated with sterilized distilled water were used as negative control [21]. The plates were incubated in a dark space at 25 °C for 48 h [48] and germination rates were monitored 24 h and 48 h post-incubation. When radicals protruded from the seed coat, seeds were considered germinated. After three days, the plates were maintained at room temperature in a Day/Night cycle (~12/12 h) for an additional 72 h and the total length, fresh and dry weight were measured [50]. The germination rate and vigor index were computed according to the following equations [51]:

- Germination rate (%) = (Number of germinated seeds/Total number of seeds) × 100
- Vigor index = Germination rate (%) × Total seedling length (cm)

#### 2.7.2. In Vivo Pot Experiment under Saline Irrigation Conditions

##### Inoculant Preparation and Seeds Treatment

Experimental studies were conducted under shade house conditions on quinoa plants using selected bacterium. *Chenopodium quinoa* seeds (*Titicaca* variety) were inoculated with the bacterial suspension as described above. We used plastic pots (height 30 cm, diameter 20 cm) containing 5 kg of a substrate composed of a sterilized mixture of horticultural sand and agricultural soil (4:1). Here, fifteen inoculated seeds were shown in each pot with four replications per treatment arranged in a complete randomized design (CRD) constructed using Pacman package in RStudio (https://www.rstudio.com/ (accessed on 10 April 2019). Then, 10 days after sowing, the seedlings were thinned to two plants per pot. At the two-leaf stage, each pot was inoculated with 20 mL of the bacterial suspension (DO_600nm_ = 0.8). Quinoa seeds treated with sterilized distilled water were used as control. Pots were watered twice a day with tap water until starting the saline irrigation treatment. Four treatments were performed in this experiment as described in Table 1.

##### Irrigation Treatment

At the five-leaf stage, the plants were watered daily with two irrigation treatments. The first set of plants was treated by non-saline irrigation (0 Mm NaCl added), while the second was irrigated with a saline solution of 400 mM NaCl (Solvachim, Casablanca, Morocco). The concentration of saline irrigation solution was progressively elevated in 100 mM increments to reach the level of 400 mM NaCl that was maintained until plant harvesting. To avoid drought stress, pots were irrigated in a way that the water ooze reached the bottom of the pots [52].

##### Plant Vegetative Attributes Measurement

Post day 45 day of growth, the leaf chlorophyll content index was recorded using a chlorophyll content meter (Hansatech instruments, Model CL-01). The next day, plants were harvested, and the soil was rinsed off from the roots under tap water. The length and weight of both roots and shoots were measured. Leaf area was determined using the *Petiole* mobile application. Dry weights of samples were recorded after drying in an oven at 70 °C for 48 h.

##### Plant Physiological Attributes Analysis

Harvested plants were used to determine P, K^+^, Na^+^, and Ca^2+^ concentrations at the agricultural innovation and technology transfer center (AITTC–UM6P) using optical emission spectrometry coupled to inductively plasma (*Agilent 5110* ICP-OES) (Santa Clara, CA, USA). The results are expressed as a percentage of dry matter (% DM). Quadruplicate sets were performed for each sample.

### 2.8. Bibliometric Analysis of Bacillus velezensis Strains

To get a panoramic view of studies carried out so far on *B. velezensis* strains, we performed data analysis to construct and visualize bibliometric networks [53]. Data were collected from the Scopus database using the TITLE-ABS-KEY search: “bacillus; AND velezensis”. All published documents were retrieved, and the co-occurrence analysis of high-frequency keywords was performed to construct a knowledge map of the main strong domain of research related to *B. velezensis*.

### 2.9. Statistical Analysis

The comparison between treatments was performed using a one-way analysis of variance (ANOVA one-way) followed by mean comparisons according to the post-hoc analysis with Dunnett’s test. Significant differences were set at *p* < 0.05.

## 3. Results

### 3.1. The QA2 Isolate Solubilizes a High Level of Phosphate

Following bacteria dilution plating on TSA, up to 79 single colonies were screened for P solubilization using qualitative assay following spot inoculation on NBRIP agar plates. Based on the solubilization halos surrounding colonies, one isolate named QA2 was selected for further studies. We noticed that the morphology of QA2 on the plate was irregular, smooth and umbonate, viscous, with a pale yellowish color.

**Figure 1:**
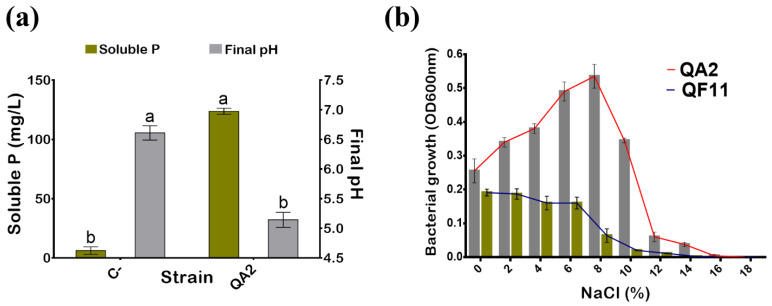
(**a**) Phosphate solubilization by the QA2 isolate in presence of 0.5% TCP in NBRIP broth and final pH of media. Uninoculated NBRIP media were used as negative control (C−). (**b**) Effect of NaCl on QA2 growth in TSB broth after 48 h of incubation. The experiment was carried out in triplicate (n = 3). *Enterobacter asburiae* QF11 was used as PGPR reference strain. Letters in superscript indicate the significant difference at 95% between treatments.

QA2 isolate exhibited a high capacity to solubilize the insoluble P (TCP) reaching 123.66 ± 2.51 mg/L (Figure 1a). In contrast, soluble P detected in the uninoculated medium did not exceed 9 mg/L. Not surprisingly given, P solubilization efficiency was negatively correlated with the final pH of the medium, which dropped from 7 to 5.14 ± 0.12 (Figure 1a).

### 3.2. The QA2 Isolate Is Halotolerant, Mesophilic, and Sensitive to Antibiotics

Salinity is abiotic stress that affects bacterial development. Here, we found that the QA2 isolate tolerated up to 11% NaCl final concentration (Table 2). Next, we performed similar experiments in a liquid TSB medium. The maximal bacterial growth of the QA2 isolate was reached at 8% NaCl final concentration. Although the growth continued to decrease to 14%, the total growth arrest was fixed at 16% NaCl (Figure 1b). QF11 (*Enterobacter asburiae*) strain, used as a PGPR control, grew at 0% NaCl but the bacterial mass proportionally decreased as salt concentration increased until the total growth inhibition at 14% NaCl.

### 3.3. The QA2 Isolate Is Endowed with High IAA Production and Exhibits a Low Zinc Solubilization Activity

Production of IAA by bacteria is considered one of the remarkable PGPR features. The QA2 isolate was assessed, in vitro, for its capacity to produce IAA in TSB medium supplemented with 0.1% L-tryptophan used as a metabolic precursor. We found that QA2 synthesized IAA with up to 43.39 ± 6.18 µg/mL while the value in uninoculated control media did not exceed 8.13 µg/mL.

Zinc is an essential micronutrient required for enzyme activation and protein synthesis in many organisms including plant tissues. Therefore, we investigated the ability of the QA2 isolate to solubilize three insoluble forms of Zn on solid media. Our results showed that ZnO was the only solubilized form as illustrated by the halos surrounding QA2 colonies (Table 2).

### 3.4. The QA2 Isolate Is a High Siderophores Producer

**Figure 2:**
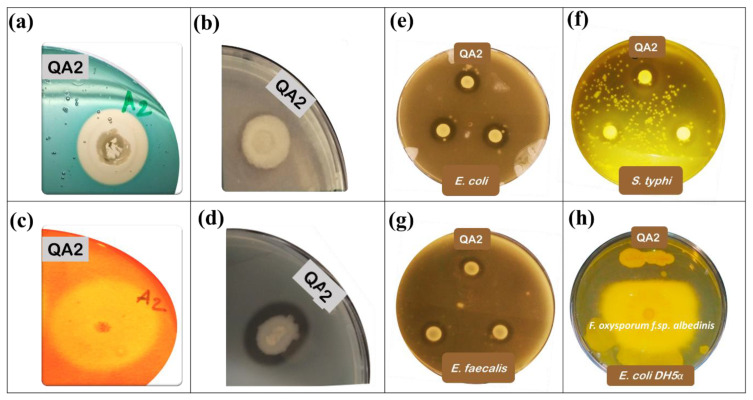
Schematic illustration showing the various tests performed on plates (Petri dishes with 9 mm in diameter) for the assessment of PGP traits of QA2 PSB. (**a**): Siderophore production, (**b**): ZnO solubilization, (**c**): Cellulase production, (**d**): Protease production, (**e**): Antibacterial activity against *E. coli*, (**f**): Antibacterial activity against *S. typhi*, (**g**): Antibacterial activity against *E. faecalis*, (**h**): Antifungal activity against *F. oxysporum f.sp. albedinis* (above) and *E. coli* DH5α (below).

Siderophores are iron chelators of dual interest; they are an important source of assimilable iron for plants and participate in spatial colonization against plant pathogens. We found that the level of siderophores production is significantly higher in the QA2 isolate compared to *Enterobacter asubiae* QF11 strain used as a reference PGPR (Table 2, Figure 2a).

### 3.5. The QA2 Isolate Is a Strong Biofilm Producer

**Figure 3:**
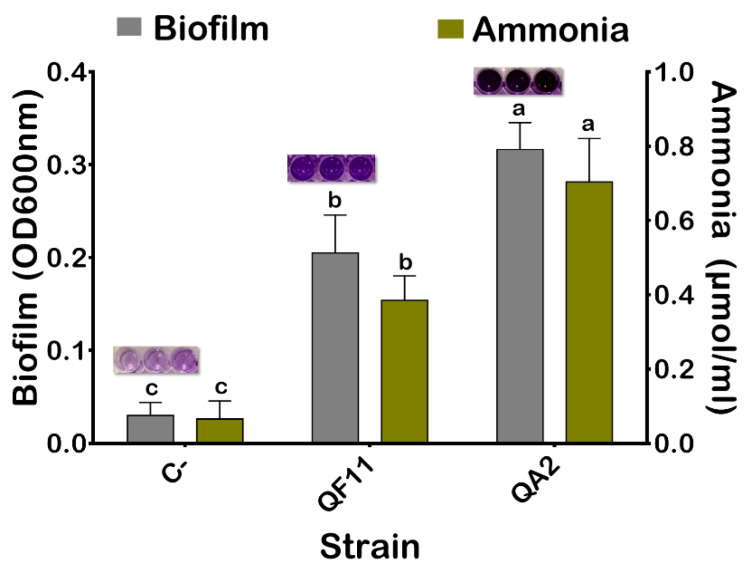
Biofilm and ammonia production by QA2. The values represent means of three replicates ± standard deviations. Uninoculated TSB media and QF11(*Enterobacter asburiae*) were used as negative (C−) and positive controls. Error bars represent standard errors for three samples from three replications. Letters in superscript indicate the significant difference to the control (C−) at 95% between treatments.

Biofilm formation by bacteria is an important phenomenon that plays numerous roles in many physiological processes [54]. As shown in Figure 3, the QA2 isolate produced a very high amount of biofilm. The obtained mean value of OD_600nm_ of the negative control was 0.03. Therefore, results less than or equal to this value were considered as the lack of biofilm formation.

### 3.6. The QA2 Isolate Produces a High amount of Ammonia and HCN and Elicited Anti-Pathogenic Bacterial Activity

Ammonia and HCN are chemical compounds producing various benefits for plant health mainly by acting as metabolic inhibitors against phytopathogens [55]. The QA2 strain was able to produce a high amount of ammonia (0.7 μmol/mL) and HCN (Figure 3 and Table 2).

Next, we investigated the antagonistic effect of our strain on selected clinical pathogenic bacteria. Remarkably, we found that QA2 showed a wide growth inhibitory spectrum against all tested pathogenic bacteria namely *S. aureus*, *S. typhi*, *E. faecalis* and *E. coli,* and against the fungus *C. albicans* (Figure 2e–g & Table 2). In contrast, no effect on pathogenic bacterial growth was seen using *Enterobacter asubiae* QF11 [23] (Results not shown).

### 3.7. The QA2 Isolate Inhibits Fungal Growth and Overproduces Extracellular Enzymes

Confrontation test on PDA plates showed that QA2 exhibited a notable antagonistic effect against the phytopathogenic fungus *F. oxysporum f.sp. albedinis.* As a control, strain *E. coli* DH5α (Negative control) was totally invaded by the fungus (Figure 2h).

Bacterial extracellular enzymes including proteases and cellulases are involved in soil fertilization and in the biocontrol of phytopathogens through the degradation of microbial membranes [56]. To investigate how QA2 could act against pathogenic bacteria, we hypothesized that it might secrete extracellular enzymes such as proteases and cellulase. To test this hypothesis, we monitored the production of protease and cellulase by the QA2 strain (Figure 2c). As for the cellulase assay, we found that the ratio “halo diameter/colony diameter” was 5.57 for QA2 in. Protease was also produced by QA2 (Figure 2d), with a diameter rate of 1.15 (Table 2). As a negative control, neither protease nor cellulase activities were detected in *E. asburiae* QF11 strain [23].

### 3.8. The QA2 Isolate Belongs to the Genus of Bacillus velezensis

**Figure 4:**
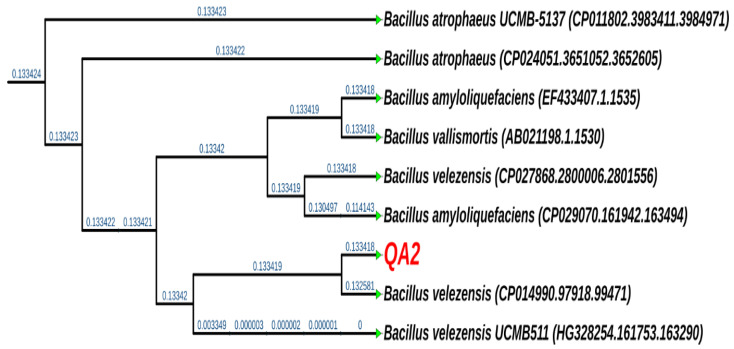
Phylogenetic tree of QA2 strain based on PHYLIP Neighbor-Joining method of the *16S rRNA* gene sequences using UGENE. The *16S rRNA* gene sequences and tree of closely related species were obtained from the SILVA database.

Phylogenetic analysis of generated DNA sequences of the strain by *16S rRNA* gene sequencing and analysis revealed that QA2 corresponds to *Bacillus velezensis* with 99% identity (Figure 4). Similarly, the sequence and analysis of the chaperonin *cpn60* gene revealed 98% identity to *Bacillus velezensis*. The *16S rRNA* gene sequence was deposited under the accession number MN809632.

### 3.9. Strain B. velezensis QA2 Stimulates Quinoa Seed Germination In Vitro

**Figure 5:**
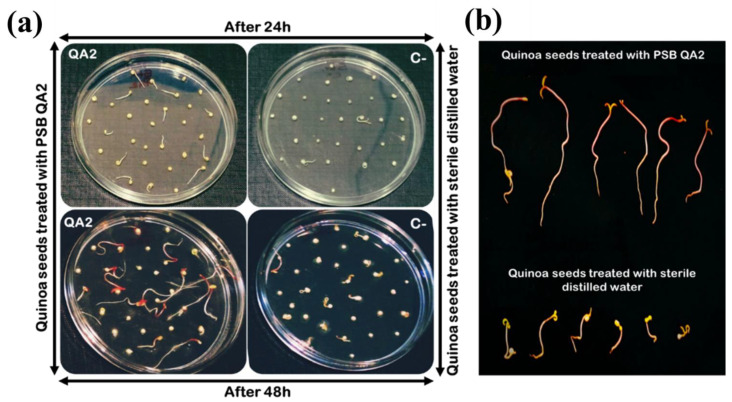
Effect of *B. velezensis* QA2 strain on early quinoa seed germination and seedlings. (**a**) Germination rates were monitored after 24 and 48 h of incubation at 25 °C. (**b**) Germinated seeds were maintained at room temperature for another 3 days and the total length of seedlings was measured at day 5.

Inoculation of seeds, soil, or plants by PGPR is a promising approach to improve global agricultural production and optimize nutrient-use efficiency [57]. The inoculation of quinoa seeds revealed that QA2 bacterial suspension exerted a significant positive influence on early plant growth namely germination rate, total length, and fresh and dry weights (Table 3 and Figure 5a,b). Synoptically, compared to uninoculated seeds, seeds coated with QA2 bacterial suspensions significantly increase the germination rate by 305%, a result that is more pronounced after 24 h of incubation at 25 °C. The same effect was recorded in the total length of seedlings, with a significant increase reaching up to 281.17%. Similarly, germinated quinoa seeds showed a 98.88% and 81.8% increase in fresh and dry weights, respectively. The resulting significant increases in these parameters resulted in a higher seedling vigor index (Table 3).

### 3.10. B. velezensis QA2 Strain Improves Shoot Biomass of Quinoa Plant Cultivated In Vivo under Salt Stress

**Figure 6:**
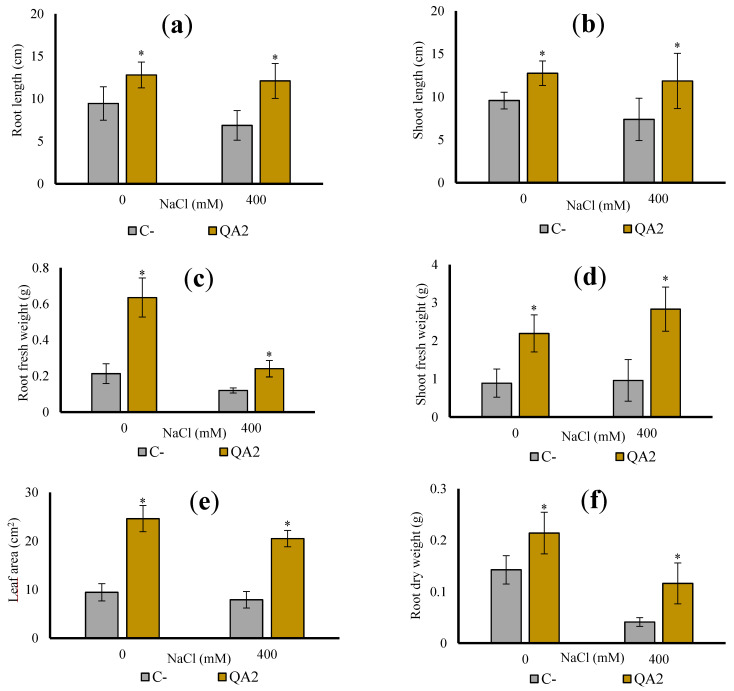

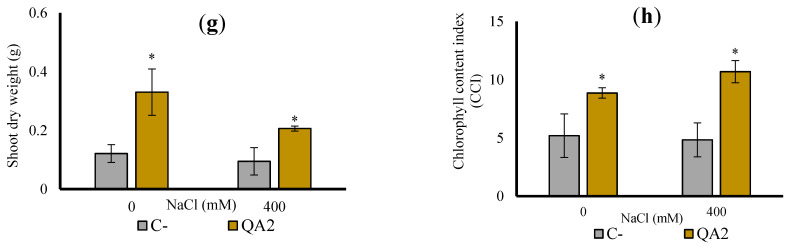
Effect of *B. velezensis* QA2 strain on quinoa plant growth parameters after 45 days of cultivation under saline conditions. (**a**) Root length, (**b**) Shoot length, (**c**) Root fresh weight, (**d**) Shoot fresh weight, (**e**) Leaf area, (**f**) Root dry weight, (**g**) Shoot dry weight, (**h**) chlorophyll content index. The values represent means of replicates (n = 4) ± standard deviations. Asterisks in superscript indicate the significant difference from the control at 95% between treatments.

Interactions of bacteria within the tripartite “plant–soil–microbes” microcosm in the rhizosphere are determinant events for their functional diversity. Therefore, the in vivo test is of considerable importance as it relatively simulates field conditions. Phenotypic growth parameters of plants recorded in the pot study are presented in Figure 6. As expected, the growth of plants inoculated with *B. velezensis* was higher growth compared to non-inoculated plants that produced less biomass. *B. velezensis* QA2 strain promoted plant growth in the absence as well as in the presence of salt stress. Saline irrigation negatively affected plant growth compared to the set irrigated by non-saline water (Figure 6). This negative influence was significantly alleviated by bacterial inoculation, which improved root fresh weight by 198.82%, the shoot fresh biomass by 146.06%, the shoot length by 134.21%, and finally the root length by 135.44% (Figure 6a–d). Dry biomass of both shoots and roots also increased under these irrigation conditions (Figure 6f,g). Moreover, when compared to the uninoculated quinoa plants, the *B. velezensis* QA2 strain improved the leaf development (i.e., leaf area) under both irrigation conditions (Figure 6e).

### 3.11. Analysis of Chlorophyll and P, Na^+^, K^+^ and Ca^2+^ Contents upon Plant Inoculation with B. velezensis QA2 Strain

To further characterize the effect of *B. velezensis* QA2 inoculation under salt or free salt treatments, we monitored the chlorophyll content of generated leaves. Our results indicate that inoculation with *B. velezensis* QA2 significantly increased the chlorophyll content index of leaves. Regarding the first salt-free treatment, QA2 was performant with an improvement reaching up to 55.43%. As for the second treatment carried out in the presence of salt stress, the chlorophyll content was increased by 120.86% following QA2 inoculation (Figure 6h).

**Figure 7:**
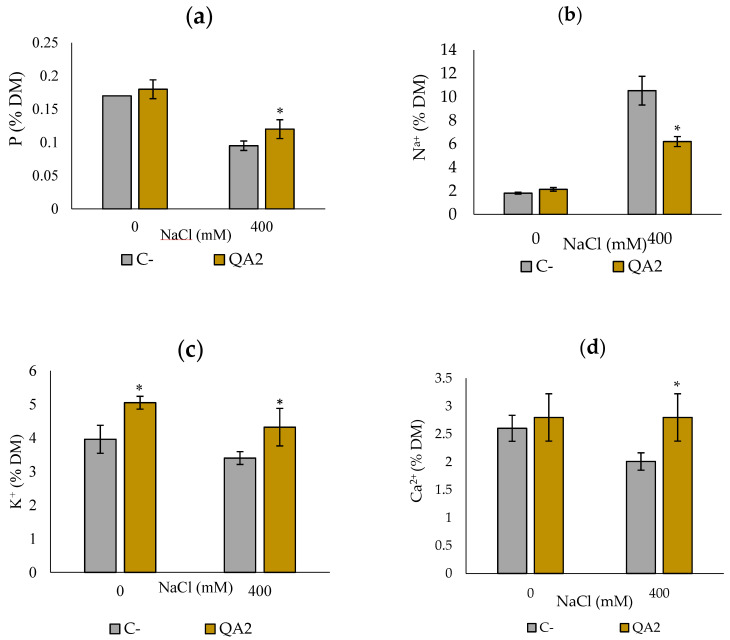
Effect of salt stress induced by NaCl and *B. velezensis* QA2 inoculation on the uptake of phosphorus (P) and ions by quinoa plants (**a**) P (**b**) Na^+^ (**c**) K^+^ (**d**) Ca^2+^. The values represent means of replicates (n = 4) ± standard deviations. Asterisks in superscript indicate the significant difference to the control at 95% between treatments.

As P content in plants is a critical growth macro-element, we analyzed its level and showed that bacterial inoculation improved P mobilization to plants under saline treatment. Upon inoculation with *B. velezensis* QA2 strain, compared to non-inoculated plants, P uptake was very high, 41.17%. In contrast, under free-saline irrigation treatment, variance analysis did not permit the detection of significant differences in all treatments compared to the negative control (Figure 7a).

To evaluate the effect of our bacteria to mitigate salt stress, we next monitored the ionic balance in sodium (Na^+^), potassium (K^+^), and calcium (Ca^2+^) ions within inoculated quinoa plants. Quinoa plants without saline irrigation increased Na^+^ accumulation and decreased K^+^ and Ca^2+^ contents. Compared to the control plants, no difference in the absorption of Na^+^ and Ca^2+^ concentrations in plants under non-saline irrigation (Figure 7b,c). However, we found that stressed plants inoculated with QA2 significantly increased K^+^ content by 27.01% and decreased Na^+^ content by 71.42% (Figure 7c). As for the Ca^2+^ accumulation, no significant differences were detected under normal irrigation, but a slight increase (8.18%) was detected in salty stressed plants inoculated by *B. velezensis* QA2 strain (Figure 7d).

## 4. Discussion

### 4.1. In Vitro and In Vivo Studies of the Bacillus velezensis QA2 Strain

Microorganisms colonizing plant root surfaces and tissues are usually nonpathogenic and beneficial for plant growth, development, and resilience. Here, we conducted a study aiming to isolate and select potent halotolerant PSB associated with quinoa plant roots. The selected QA2 strain solubilized phosphates both on plates and in liquid media (123.66 mg/L) and grew in a medium amended with 11% NaCl (Figure 1). For many years, ‘*B. subtilis* species complex’ including a closely related species *B. amyloliquefaciens*, *B. licheniformis*, *B. pumilus*, and *B. subtilis* were difficult to classify based on classic taxonomical features such as morphology, physiological traits, guanine-cytosine content, and *16S rRNA* gene sequencing [58]. In this study, QA2 genotyping identification was based on both *16S rRNA* and *cnp60* genes sequencing analysis, which enabled us to assign QA2 to *Bacillus velezensis* genus. Moreover, it has been shown recently that *B. amyloliquefaciens subsp. plantarum* and *B. methylotrophicus* belong to the same phylogenetic group and are synonymous, and successively, *B. methylotrophicus* and *B. velezensis* are also synonymous due to the high phenotypic and genotypic taxa coherence [59].

Considering the variation of PSB populations and their functional diversity, it is extremely important to search for PGPB with multiple beneficial properties. *Bacillus velezensis* QA2 was therefore assessed in vitro for plant-growth-promoting proprieties through a range of assays including IAA production, siderophore release, antibacterial activity, production of ammonia, HCN, biofilm, and extracellular enzymes. In addition to being the first class of phytohormones to be identified, IAA has been for a long time used as an important line for selecting beneficial soil bacteria with a strong ability to promote plant growth [60]. IAA could influence virtually all physiological aspects of plant development [61]. Our strain *B. velezensis* QA2 produced around 50 µg/mL of IAA. This finding is not surprising as up to 80% of rhizobacteria are IAA producers [62]. It is also extremely important to point out that the capacity of bacteria to synthesize IAA depends on the tryptophan concentrations and on its availability in the rhizosphere [63]. Mohammad Sayyar et al. (2020) have shown that IAA production by *B. velezensis* Lle-9 is positively correlated to tryptophan concentrations with IAA levels ranging from 23.2 ± 1.9 μg/mL to 165.7 ± 5.8 μg/mL at tryptophan concentrations ranging from 2 mg/mL to 6 mg/mL, respectively [64]. Here, our strain *B. velezensis* QA2 produced up to 50 µg/mL of IAA in the presence of 0.1% L-tryptophan.

In agriculture, iron chelation by siderophores is a desired property of dual interest; on one hand, bacterial siderophores are an important source of assimilable iron for plants; on the second hand, iron chelation by PGPR is a competitive trait for spatial colonization against phytopathogens [65]. We revealed that the *B. velezensis* QA2 strain secretes these chelators as judged by the width of the halo surrounding colonies (Figure 2a). Siderophores may also act as stimulatory signals to produce other metabolites involved in the control of pathogens [66]. They chelate critical concentrations of heavy metals and solubilize minerals which consequently stimulates plant nutrition and growth [67]. It is also well established that siderophores have an obvious potential role in enhancing P availability [68]. Besides the role of siderophores in neutralizing the harmful effects caused by phytopathogenic microorganisms, either Hydrogen cyanide (HCN), a volatile metabolite with antagonistic vocation, or ammonia are toxic for pathogens growth and fungal activity by acting as metabolic inhibitors [69]. We showed that *B. velezensis* QA2 overproduced HCN and ammonia. Comparatively, several studies highlighted the role of HCN and ammonia-producing bacteria, such as *Pseudomonas* strains, in the biocontrol of a wide range of fungi-plant diseases [70,71]. Besides this, nitrogen (N) and its derivative ammonia promote plant growth as they provide N directly to plant [72].

A plant’s rhizosphere is an environment that is nutritionally and physiochemically beneficial for root microflora due to the abundance of organic molecules produced by plants. Some of them are chemical signals that attract motile bacteria to the root (i.e., chemotaxis), thereby favoring biofilm formation [73]. Production of biofilm favors adhesion to roots and plant colonization due to the secretion of extracellular polymeric substances (EPS) composed of proteins, nucleic acids, polysaccharides, and lipids. EPS mainly function to facilitate cell stability, adhesion, and interconnection to ensure plant stability and protection from harsh environmental conditions, infectious microbes, and nutrient centralization [74,75]. We demonstrated here that *B. velezensis* QA2 strain induced biofilm formation. For instance, bacterial EPS, by binding to cations, could alleviate salt stress by restricting Na^+^ uptake by roots [64].

We demonstrated that *B. velezensis* QA2 produced two important enzymes: protease and cellulase, considered as important mechanisms involved in plant growth promotion [76,77] by playing a key role in the biological and physicochemical transformations of soils [78]. Both enzymes are involved in the biocontrol of pathogens through the degradation of fungal membranes [56].

The search for bacteria having a broad spectrum of antimicrobial activity while being less aggressive for the plant still seems ideal. Many strains of the *B. velezensis* genus have been shown to inhibit the growth of pathogenic bacteria [58], fungi [79], and nematodes [80]. The remarkable inhibitory of our strain *B. velezensis* QA2 of different pathogenic strains strongly suggest its use as a powerful PGPB of wide biocontrol spectrum [81]. This is likely mediated through the secretion of an array of secondary metabolites [82], and by the induction of systemic resistance in the host plant [83]. The reported antibacterial effect of *B. velezensis* strains has been also linked to the production of cyclic lipopeptide compounds such as iturin, fengycin, bacillomycin-D [84]. In addition, surfactin, and polyketides such as macrolactin, difficidin, and bacillaene [85] are considered active molecules against the phytopathogenic bacteria *Ralstonia solanacearum* and the fungi *Fusarium. oxysporum* [81]. Recently, *B. velezensis* PEA1 was reported to inhibit the growth of *Fusarium oxysporum* and to elicit a systemic resistance against both fungal and viral infections more likely via the production of pyrrolo (1,2-a) pyrazine-1,4-dione [86].

In the present work, we showed that the *B. velezensis* QA2 strain supported up to 11% NaCl final concentration on plates but only 8% NaCl in liquid media. This discrepancy between the two media is probably due to the easier salt access of bacteria in the liquid media. To fight biotic stresses such as salinity, plants activate several physiological responses including phytohormones synthesis, antioxidant production, and nutrient uptake regulation. Furthermore, plant-associated bacteria could be a considerable boost for plant growth under such harsh conditions via the release of growth-promoting substances and regulators [14,87].

The inoculation of quinoa plants by *B. velezensis* QA2 improved plant growth with a significant increase in vegetal biomass (Roots/Shoots) and P uptake. We also detected an increase in CCI of leaves under saline conditions, which could be explained by the capacity of our isolate to accumulate nitrogen compounds such as amino acids and polyamines [88].

It is well documented that salinity results from a deficiency in nutrient uptake and induction of Na^+^ accumulation in plant tissues. Consequently, this causes devastating effects on plant physiology including photosynthesis inhibition, reactive oxygen species (ROS) formation, inhibition of protein synthesis, inactivation of enzymes, and alteration of cellular integrity [89]. Experimental findings indicate that PGPR can help plants successfully adapt to different stressful situations [90]. This could result in an increase in nutrient uptake in the host plant [91]. This increase may be accentuated especially in salt-exposed crops because soil salinity significantly reduces nutrient assimilation [91]. Comparatively, a previous study reported that inoculation of wheat plants with phosphate-solubilizing and phytohormone-producing bacterial strains such as *Azospirillum, Bacillus,* and *Enterobacter* improves growth and grain yield [92]. Likewise, our strain showed in vitro positive PGP traits, such as P solubilization and IAA, HCN, ammonia, biofilm, and siderophore production, which help plants to better absorb nutrients. Here, we showed that *B. velezensis* QA2 promoted the P and Ca^2+^ assimilation under salty stress but not significantly in non-stressful conditions. This suggests that *B. velezensis* QA2 triggered salt stress response in plants through alternative mechanisms than conventional PGP traits.

Comparatively, using the tomato plant model, the endophytic halotolerant *B. velezensis* FMH2 isolated from the Sfax solar saltern (Tunisia) was shown to improve plant biomass under normal and salty conditions (up to 171 mM NaCl). This effect was accompanied by an improvement of the photosynthetic and antioxidant parameters as well as a decrease in toxic ions uptake [93]. Moreover, the ability of *B. amyloliquefaciens* SQR9 to mitigate salt stress effects on maize was conferred through maintaining photosynthesis processes and protecting plants from ROS damage, Na^+^ toxicity, as well as from osmotic stress [94]. The low Na^+^ content in shoots and roots was explained by hampering sodium ions accumulation through the root refusing or expelling of these ions. Noteworthily, they proved that in the absence of stress conditions, SQR9 did not significantly promote plant growth. This observation rules out the possibility that *B. amyloliquefaciens* SQR9 induces plant salt tolerance by promoting plant growth. Other studies reported that the application of PGPB triggered antioxidant defense mechanisms in host plants and induced antioxidant enzyme syntheses such as that of peroxidase (POX), superoxide dismutase (SOD), and catalase (CAT) [95,96]. *B. velezensis* QA2 may likely act by maintaining plant membrane integrity, reducing lipid peroxidation, and increasing antioxidant production. Collectively, our results support the use of *B. velezensis* QA2 strain as a potential candidate to mitigate arable soil salinity and as a biofertilizer.

### 4.2. Hot Topics Analysis of Bacillus velezensis Strains

**Figure 8:**
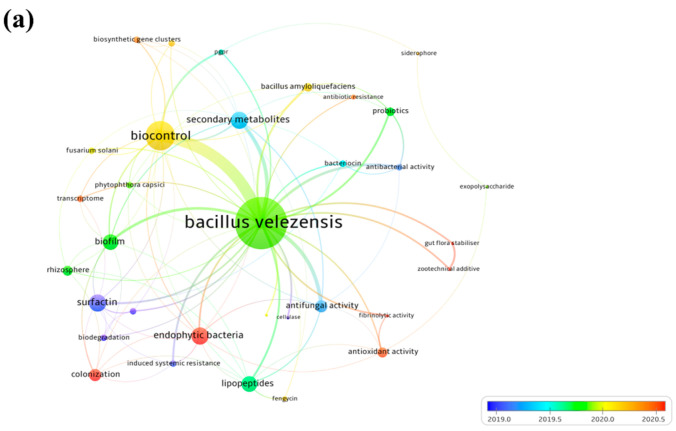

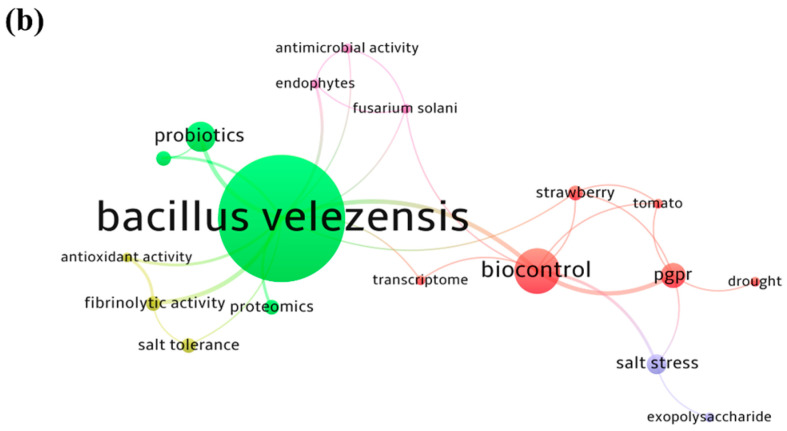
Bibliometric analysis of documents published on *B. velezensis* strains according to Scopus database. The TITLE-ABS-KEY search was used for specific keywords “*bacillus*; and *velezensis*”. (**a**) Co-occurrence map visualization and (**b**) Knowledge map of *B. velezensis*. Each term is represented by a circle whose size is indicative of the intensity of the research activity.

At the time of writing, we performed a bibliometric data analysis to give a panoramic view of the different studies carried out on *B. velezensis* strains. Data were collected from the Scopus database using the TITLE-ABS-KEY search: “*bacillus*; AND *velezensis*”. A total of 835 documents were retrieved and the co-occurrence analysis of high-frequency keywords was performed to construct a knowledge map of the main strong domain of research related to *B. velezensis.* In the visualization results, each keyword is represented by a circle. From the analysis of Figure 8a, three main topic groups were identified, which are “the secondary metabolites produced”, “the biological activities”, and “the neighboring strains”. The field of PGP activities and biocontrol are strongly connected to metabolite production such as that of lipopeptides. After further domain structure detection as shown in Figure 8b, the main topic in the field of tolerance to salt stress induced by *B. velezensis* was “Exopolysaccharides”. These two terms (salt stress and exopolysaccharide) are clustered within the same group (Circles in blue). Moreover, salinity tolerance is also linked to the “antioxidant and fibrinolytic activities” (Circles in yellow). Hence, the induced salt tolerance in plants using *B. velezensis* as an inoculant could be closely linked to these activities. This analysis revealed the relationship between *B. velezensis*-related keywords and provide a comprehensive outlook of the current research on this species.

## 5. Conclusions

In this study, we demonstrated that quinoa-associated halotolerant P solubilizing *B. velezensis* QA2 strain is endowed with a promising plant growth-promoting potential in both in vitro and in vivo studies under either normal or saline conditions. Interestingly, we demonstrated that *B. velezensis* QA2 exhibited, in addition to the PGP traits, a remarkable biocontrol activity against common human and plant bacterial and fungal pathogens. *B. velezensis* QA2 PGP attributes and antagonistic features are related to its biochemical and physiological characteristics and to its secondary metabolites. The present study opens a new avenue for further investigating the underpinning mechanisms involved by *B. velezensis* QA2 to mitigate salt stress. Our next challenge will be to identify the nature of biomolecules produced and/or secreted by *B. velezensis* growth under various induced stresses. Lastly, many *Bacillus* strains are considered safe for human consumption and are awarded GRAS status (Generally Regarded as Safe) by the Food and Drug Administration (FDA). In-lane with this finding, we revealed here that our *B. velezensis* QA2 strain is sensitive to frequently encountered antibiotics and could represent a safe biotechnological commercial inoculant to be applied as a biocontrol and biofertilizer agent suitable for salty soils.

## Figures and Tables

**Table 1:** Pot experiment work plan.

0 mM NaCl		400 mM NaCl	
Symbol	Treatment	Symbol	Treatment
C−	Seeds treated with sterilized distilled water (Negative control)	C−	Seeds treated with sterilized distilled water (Saline control)
QA2	Seeds treated with QA2 isolate	QA2	Seeds treated with QA2 isolate

**Table 2:** Summary of some morphological, physiological, and biochemical characters of the QA2 isolate using plate assays.

Assay	Method	Result
Bacterial isolate		QA2
Morphology	Culture on TSA plate at 30 °C	Motile
		Velvety
		Rod-shaped
Extreme growth properties	NaCl tolerance (%)	11%
	Maximum tolerable temperature	45 °C
Siderophore production	CAS medium	+++
HCN production	TSA medium amended with 0.44% glycine	+++
Extracellular enzymes (*Halo diameter/colony diameter*)	Protease	1.15
	Cellulase	5.41
Zinc solubilization	ZnO	++
	Zn_3_(PO_4_)_2_	−
	ZnCO_3_	−
Antibiotic resistance using antibiotic-amended agar	Ampicillin (100 µg/mL)	−
	Chloramphenicol (20 µg/mL)	−
	Streptomycin (100 µg/mL)	−
	Spectinomycin (60 µg/mL)	−
	Kanamycin (50 µg/mL)	−
	Tetracycline (10 µg/mL)	−
Antibacterial activity using disk diffusion method	*Escherichia coli*	+++
	*Salmonella typhi*	+++
	*Enterococcus faecalis*	++
	*Staphylococcus aureus*	+++
Antifungal activity using bacteria—fungi confrontation assay	*Fusarium oxysporum f.sp. albedinis* *Candida albicans*	+++++

The ‘+’ and ‘−’ signs indicate efficiencies as follow: −, negative result; +, weakly positive; ++, moderately positive; +++, highly positive.

**Table 3:** Effect of *B. velezensis* QA2 strain on quinoa seed germination in vitro.

Germination Parameters		C−	*B. velezensis* QA2
Germination rate (%)	24 h	16.6 ± 6.65	57.3 ± 10.26 *
	48 h	58.6 ± 5.13	89.66 ± 6.5 *
Total Length (cm)		1.7 ± 0.55	6.48 ± 0.87 *
Fresh Weight (mg)		36 ± 10.5	71.6 ± 6.11 *
Dry Weight (mg)		6.6 ± 1.52	12 ± 2.64 *
Seedling Vigor Index		99.46 ± 56.95	580.60 ± 79.06 *

C− (Negative control), seeds treated with sterile distilled water. The numerical values represent means of replicates (n = 3) ± standard deviations. Asterisks in superscript indicate the significant difference to the control at 95% between treatments.

## Data Availability

This study did not report any data.
